# Mechanical force-activated CD109 on periodontal ligament stem cells governs osteogenesis and osteoclast to promote alveolar bone remodeling

**DOI:** 10.1093/stcltm/szae035

**Published:** 2024-06-17

**Authors:** Yang Li, Yi Li, Chao Liu, Xinyi Yu, Ziqi Gan, Lusai Xiang, Jinxuan Zheng, Bowen Meng, Rongcheng Yu, Xin Chen, Xiaoxing Kou, Yang Cao, Tingting Ai

**Affiliations:** Hospital of Stomatology, Guangdong Provincial Key Laboratory of Stomatology, Guanghua School of Stomatology, Sun Yat-sen University, Guangzhou 510055, People’s Republic of China; Hospital of Stomatology, Guangdong Provincial Key Laboratory of Stomatology, Guanghua School of Stomatology, Sun Yat-sen University, Guangzhou 510055, People’s Republic of China; Hospital of Stomatology, Guangdong Provincial Key Laboratory of Stomatology, Guanghua School of Stomatology, Sun Yat-sen University, Guangzhou 510055, People’s Republic of China; Hospital of Stomatology, Guangdong Provincial Key Laboratory of Stomatology, Guanghua School of Stomatology, Sun Yat-sen University, Guangzhou 510055, People’s Republic of China; Hospital of Stomatology, Guangdong Provincial Key Laboratory of Stomatology, Guanghua School of Stomatology, Sun Yat-sen University, Guangzhou 510055, People’s Republic of China; Hospital of Stomatology, Guangdong Provincial Key Laboratory of Stomatology, Guanghua School of Stomatology, Sun Yat-sen University, Guangzhou 510055, People’s Republic of China; Hospital of Stomatology, Guangdong Provincial Key Laboratory of Stomatology, Guanghua School of Stomatology, Sun Yat-sen University, Guangzhou 510055, People’s Republic of China; Hospital of Stomatology, Guangdong Provincial Key Laboratory of Stomatology, Guanghua School of Stomatology, Sun Yat-sen University, Guangzhou 510055, People’s Republic of China; Hospital of Stomatology, Guangdong Provincial Key Laboratory of Stomatology, Guanghua School of Stomatology, Sun Yat-sen University, Guangzhou 510055, People’s Republic of China; Hospital of Stomatology, Guangdong Provincial Key Laboratory of Stomatology, Guanghua School of Stomatology, Sun Yat-sen University, Guangzhou 510055, People’s Republic of China; Hospital of Stomatology, Guangdong Provincial Key Laboratory of Stomatology, Guanghua School of Stomatology, Sun Yat-sen University, Guangzhou 510055, People’s Republic of China; South China Center of Craniofacial Stem Cell Research, Hospital of Stomatology, Sun Yat-sen University, 74 Zhongshan 2 Road, Guangzhou 510080, People’s Republic of China; Hospital of Stomatology, Guangdong Provincial Key Laboratory of Stomatology, Guanghua School of Stomatology, Sun Yat-sen University, Guangzhou 510055, People’s Republic of China; Hospital of Stomatology, Guangdong Provincial Key Laboratory of Stomatology, Guanghua School of Stomatology, Sun Yat-sen University, Guangzhou 510055, People’s Republic of China

**Keywords:** bone, cell-surface markers, cell signaling, stem cells, monocyte

## Abstract

Mechanical force-mediated bone remodeling is crucial for various physiological and pathological processes involving multiple factors, including stem cells and the immune response. However, it remains unclear how stem cells respond to mechanical stimuli to modulate the immune microenvironment and subsequent bone remodeling. Here, we found that mechanical force induced increased expression of CD109 on periodontal ligament stem cells (PDLSCs) in vitro and in periodontal tissues from the force-induced tooth movement rat model in vivo, accompanied by activated alveolar bone remodeling. Under mechanical force stimulation, CD109 suppressed the osteogenesis capacity of PDLSCs through the JAK/STAT3 signaling pathway, whereas it promoted PDLSC-induced osteoclast formation and M1 macrophage polarization through paracrine. Moreover, inhibition of CD109 in vivo by lentivirus-shRNA injection increased the osteogenic activity and bone density in periodontal tissues. On the contrary, it led to decreased osteoclast numbers and pro-inflammatory factor secretion in periodontal tissues and reduced tooth movement. Mechanistically, mechanical force-enhanced CD109 expression via the repression of miR-340-5p. Our findings uncover a CD109-mediated mechanical force response machinery on PDLSCs, which contributes to regulating the immune microenvironment and alveolar bone remodeling during tooth movement.

## Graphical Abstract

**Graphical Abstract:**
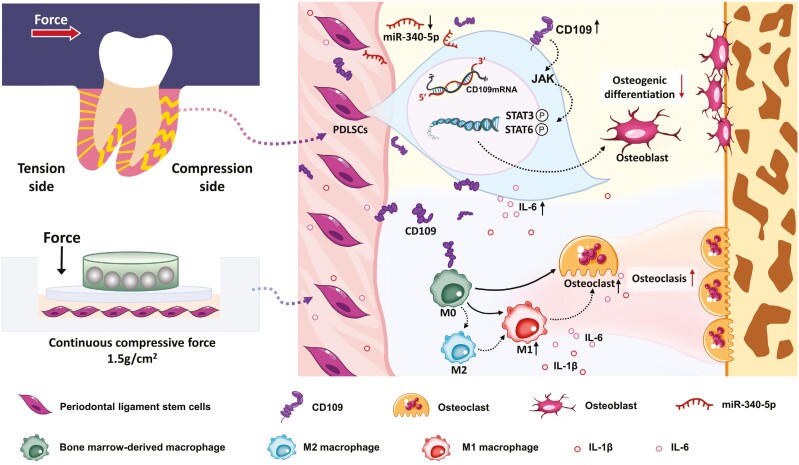

**Significance statement**

Mechanical force-mediated bone remodeling is crucial for various physiological and pathological processes. Mesenchymal stem cells can respond to mechanical stimuli to modulate bone turnover. However, the mechanisms remain unclear. Here, we found CD109 localized on the surface of periodontal ligament stem cells were sensitive to mechanical force and played an important role in activating alveolar bone remodeling. This CD109-mediated mechanical force response machinery in periodontal ligament stem cells depends on the repression of miR-340-5p, which provides a novel insight into the mechanisms of force-involved bone remodeling and the therapeutic strategies for bone metabolic diseases.

## Introduction

Mechanical force-mediated bone remodeling is critical to various physiological and pathological processes and involves multiple factors, such as stem cells and immune response. Orthodontic tooth movement (OTM) represents a distinctive process of bone remodeling triggered by mechanical force.^1,2^ This process relies on the periodontal ligament (PDL), a soft connect tissue between the root of the tooth and the alveolar bone. Due to the masticatory function of teeth, PDL is exposed to a mechanically stimulated environment, serving as a buffer and maintaining tissue homeostasis. In addition, it is also indispensable for tooth movement when loaded by exogenous mechanical loading. During this process, PDL produces a variety of inflammation-associated components to regulate bone resorption and formation.^3-5^ Periodontal ligament stem cells (PDLSCs), the main MSCs in the PDL, present the potential for self-renewal, non-specialization, multilineage differentiation, and immunoregulatory.^6,7^ It can respond to mechanical stimulation and be involved in a complex, aseptic, and inflammatory immune reaction that activates bone turnover during bone reconstruction processes.^3,8,9^ However, the detailed mechanisms remain unclear.

Cell-surface CD109, a glycosylphosphatidylinositol (GPI)-linked glycoprotein, is identified as a TGF-β co-receptor and negative regulator of the TGF-β signal pathway.^10^ Recently, CD109 has been demonstrated to express on various types of cells, like CD34^+^ positive bone marrow mononuclear cells, activated T lymphoblasts, activated platelets, endothelial cells, and several human tumor cell lines, and relate to various biological processes and diseases.^11,12^ Notably, several studies showed that CD109 was expressed on osteoblasts and osteoclasts, suggesting that CD109 may play a crucial role in bone metabolism through experiments in vivo and in vitro.^12,13^ Moreover, CD109 also exhibited the property of regulating inflammatory response toward osteoclasts, contributing to bone destruction in rheumatoid arthritis (RA).^14^

In addition, our previous study found that human PDLSCs expressed higher levels of CD109 than dental pulp stem cells (DPSCs) and dental follicle progenitor cells and exhibited differential osteogenic ability compared to the other 2 dental stem cell lines, indicating the relationship between CD109 and osteogenesis.^15^ However, the underlying molecular mechanism needs further exploration.

In this study, we used an animal model of mechanical load-induced tooth movement and an in vitro experiment involving continuous compressive force to investigate how CD109 as a mechanical transducer of PDLSCs contributes to immune response, alveolar bone remodeling, and tooth movement.

## Materials and methods

### Animal models with mechanical stimulation

All animal experiments were approved by the Ethics Committee of SUN-YAT-SEN University (SYSU-IACUC-2023-001392), and all procedures followed the Laboratory Animal Protection Law. To set up an OTM animal model, orthodontic nickel-titanium coiled springs (wire size 9 mm; diameter 0.012 inch) were ligated between the left maxillary first molar and the incisors of the 8-week-old Sprague-Dawley (SD) rats (male, weighing 150 ± 10 grams) with force applied of 60 grams, after which a flowable restorative resin (3M ESPE) was used to fix the spring. Based on previous studies, it has been indicated that the application of a 60-gram force during orthodontic tooth movement in rats is both effective and safe.^3,8^ Rats were randomly divided into 4 groups: 1 (control), 2 (force), 3 (shRNA), and 4 (shRNA + Force). The mechanical force was applied to 2 groups of rats for 10 days, as previously described. To assess the impact of CD109 on OTM, a lentiviral vector containing a short-hairpin RNA (LV-shRNA) of CD109 was injected into 3 specific sites within the PDL of the left maxillary first molars in rats belonging to both shRNA and shRNA + force groups, commencing 7 days before tooth movement. Each site received a 30 µL LV-shRNA CD109 (18 × 10^6^ TU) injection every 3 days (Supplementary Methods). After 10 days of tooth movement, the rats in each group were sacrificed for further research.

### Application of mechanical compressive force

The culture of hPDLSCs is described in the Supplementary Material. hPDLSCs were seeded separately in 6-well plates at approximately 2.5 × 10^4^. When the cell confluence reached approximately 80%, a continuous compressive force of 0.5-2.0 g/cm^2^ was applied to the hPDLSCs for 24 hours. Previous research has demonstrated that forces below 0.5g/cm^2^ have minimal impact on cells, while forces exceeding 3g/cm^2^ would lead to cell rupture and death due to excessive compression.^16,17^ The force was achieved by utilizing layers of glass and plastic tube caps with weighted metal balls, following a modified method previously described.^4^ Cells without force stimulation were used as the control group in this study. The culture medium derived from hPDLSCs, with or without mechanical loading, was collected for subsequent experiments.

### Inhibition and over-expression of CD109 on hPDLSCs

To knock down CD109, the CD109-small interfering RNA (siRNA) and negative control (NC) were designed and synthesized by Guangzhou RiboBio Company. The sequences are listed in Supplementary Table S1. To over-express CD109, the CD109 cDNA targeting sequence was cloned by PCR and was inserted into pcDNA3.1-3xFlag-T2A-EGFP vectors (PUZONGene), and empty vectors were used as negative control (NC). The cDNA targeting sequences are listed in Supplementary Table S2. When cells reached 70%-80% confluence, the transfection (siRNA: 50 μM, cDNA: 10 ng/μL) was executed with Lipofectamine 3000 (Invitrogen) according to manufacturer’s protocol. We transfected the cells once every 4 days during osteogenic induction. Transfection efficiencies were detected by Western blot.

### Micro-computed tomography analysis

The rats were sacrificed after the mechanical force was applied for 10 days. The maxillae were removed for scanning, which used a micro-computed tomography (micro-CT) system (voltage: 85 kV, current: 200 μA, resolution ratio: 10 μm; SkyScan 1276; Bruker, Germany). CTAn and CTvox software (Bruker) were used to reconstruct and analyze the radiographs. The region of interest selected for analysis was the pressure side alveolar bone of the mesial root of the maxillary first molar to determine trabecular bone volume fraction (BV/TV), trabecular thickness (Tb.Th), trabecular separation (Tb.Sp), and bone mineral density (BMD). OTM distance was assessed by 3 trained researchers, who were unaware of the group assignment, measuring the distance between 2 points (the midpoint of the distal-marginal ridge of the first molar and the midpoint of the mesial-marginal ridge of the second molar). The average value from 3 measurements was calculated to indicate tooth movement.

### In vitro osteogenesis assays

After the transfections and osteogenic induction of hPDLSCs, the alkaline phosphatase (ALP) assay was performed with Alkaline Phosphatase Stain Kit (YEASEN) conducted on day 7 as previously described^18^ and evaluated by microscopy (Carl Zeiss). After that, the cells were lysed in a 1% Triton X-100 solution for 1 hour to quantitatively analyze the ALP levels. The supernatant was collected after low-speed centrifugation (211 g, 5 minutes). The Alkaline phosphatase kit was applied to detect the supernatant, and absorbance at 520 nm was calculated by spectrophotometer (BioTek). Calcium accumulation was evaluated by Alizarin red staining after 21 days of the costimulation mentioned above. Cells were stained with 1% Alizarin Red solution (Cyagen) for 30 minutes. Following 3 rinses with deionized water, Alizarin Red-positive cells were observed using the Zeiss Axio Observer microscope.

### In vitro osteoclastogenesis assays

The culture of bone-marrow-derived macrophages (BMDMs) is described in the Supplementary Material. The co-incubated supernatants were collected from the “Culture of human PDLSCs and the application of mechanical compressive force” section and used as the macrophage-conditioned medium for subsequent osteoclastogenesis experiments. BMDMs (1.5 × 10^6^ cells/well) were cultured in a costimulation medium containing an osteoclast-inducing solution [20 ng/mL − macrophage colony-stimulating factor (M-csf) and 40 ng/mL − Receptor Activator of Nuclear Factor-κB Ligand (Rankl)] along with the macrophage-conditioned medium treated differently (in equal proportions). After 7 days of culturing, Western blot analysis was conducted to determine the presence of proteins associated with osteoclasts. Besides, as previously mentioned, the cells were stained by a TRAP staining kit (Sigma-Aldrich).

### Quantitative real-time polymerase chain reaction

The total RNA from the cells was extracted using RNAzol reagent (Yishan Biotech), and reverse transcription was carried out using the PrimeScript RT Master Mix (Takara). Quantitative real-time PCR analysis was conducted on an ABI QuantStudio5 instrument with the AceQ qPCR SYBR Green Master Mix (Yeasen). The cDNA of the microRNA was synthesized utilizing a miRNA cDNA Synthesis Kit (Ribobio), and quantification of gene expression was performed via qPCR using a miRNA qPCR Assay Kit (Ribobio). β-Actin or U6 was used as an internal control, with the primer sequences described in Supplementary Table S3.

### MicroRNA(miR) transfection

The hPDLSCs were either untreated or transfected with miR-340-5p mimics and mimics NC (RiboBio) using Lipofectamine 3000 (Invitrogen). In brief, separate mixtures of miR-340-5p mimics (100 nM), mimics NC (100 nM), and diluted Lipofectamine 3000 were prepared at room temperature for 20 minutes. Transfection was evaluated under a fluorescence microscope at 12 hours. The mixtures were then cultured with cells for 48 hours. The sequences of miR-340-5p mimics were: forward primer 5ʹ-UUAUAAAGCAAUGAGACUGAUU-3ʹ and reverse primer 5ʹ-AAUCAGUCUCAUUGCUUUAUAA-3ʹ

### Statistical analysis

SPSS 20.0 was used to conduct statistical analysis. The data were presented as means ± SD. One-way ANOVA followed by Tukey’s multiple comparisons in Prism software (version 9.0, GraphPad) was used to assess all the data. Statistical significance was considered when *P* < .05.

## Results

### Compressive force activates the expression of CD109 on PDLSCs in vivo and in vitro

**Figure 1.**
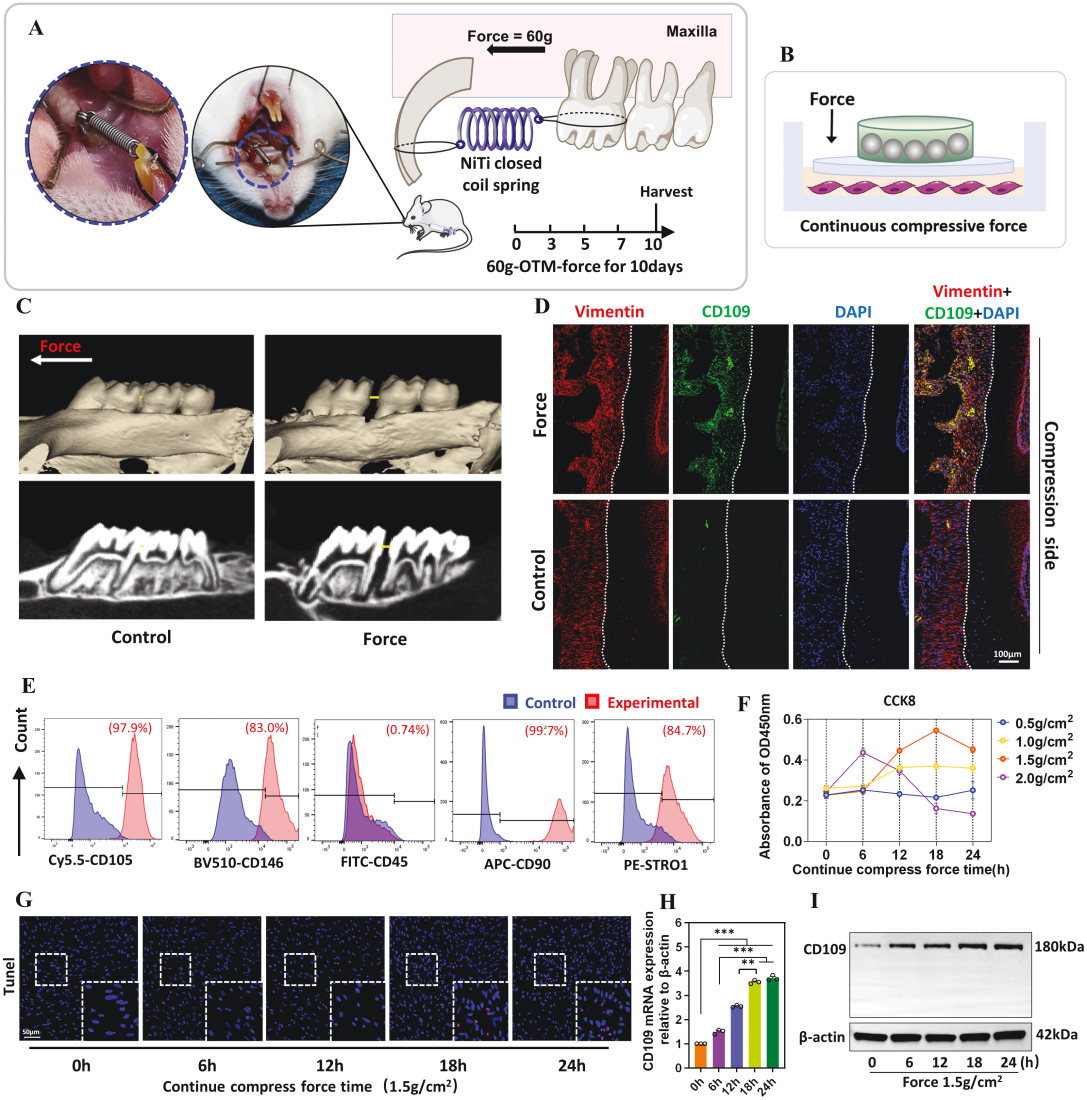
Mechanical force activated CD109 expression on PDLSCs both in vivo and in vitro. (A-B) Schematic illustration of mechanical force-induced tooth movement SD rat model and in vitro continuous compressive force model of hPDLSCs. (C) Representative micro-CT images of tooth movement for 10 days in rats. The arrow indicates the direction of mechanical force. The line segment represents the tooth movement distance. *N* = 8. (D) Representative immunofluorescence double-staining of CD109 and Vimentin in the compression side of roots. *N* = 8. (E) Flow cytometry indicated that hPDLSCs expressed high levels of mesenchymal stem cell markers CD105, CD146, CD90, and STRO1, while they were negative for hematopoietic markers CD45 on P3 hPDLSCs. *N* ≥ 3. (F) Representative CCK-8 assay of hPDLSCs after loading continuous compressive force ranging from 0.5 g/cm^2^ to 2.0 g/cm^2^ for 24 hours. *N* ≥ 3. (G) Representative Tunel assay of hPDLSCs after loading a continuous compressive force of 1.5 g/cm^2^ for 24 hours. *N* ≥ 3. (H-I) Representative qRT-PCR and Western blots of the CD109 expression on hPDLSCs. *N* ≥ 3, *P < .05; **P < .01; ***P < .001, Values are means ± SD.

We established an animal model of mechanical force-induced tooth movement (Figure 1A and 1C), the immunofluorescence staining results showed that the CD109/Vimentin double-positive cells were significantly increased in the compressive side of PDL following 10-day-force treatment (Figure 1D), while no significant changes were observed in the tension side (Supplementary Figure S1). Notably, as Vimentin is a mesenchymal stem cell marker, the results suggested a direct correlation between compressive force and CD109 biogenesis on PDLSCs in vivo. To confirm this phenomenon, hPDLSCs were isolated from the human premolar teeth and subjected to static compressive force in vitro (Figure 1B). Flow cytometric analysis identified that hPDLSCs expressed high levels of mesenchymal stem cell markers CD105 (97.9%), CD146 (83.0%), CD90 (99.7%), and STRO1 (84.7%) while were negative for hematopoietic markers CD45 (0.74%) (Figure 1E). The CCK-8 results showed that cell viability remains stable after exposure to a compressive force of 1.5 g/cm^2^ for 24 hours, and the Tunel assay indicated no significant difference in cell apoptosis (Figure 1F and G). The qRT-PCR and Western blot results showed a significant positive correlation between the expression level of CD109 on hPDLSCs and the compressive force loading time (Figure 1H and I).

### CD109 inhibits the osteogenic ability of PDLSCs through the JAK-STAT3 signaling pathway

**Figure 2.**
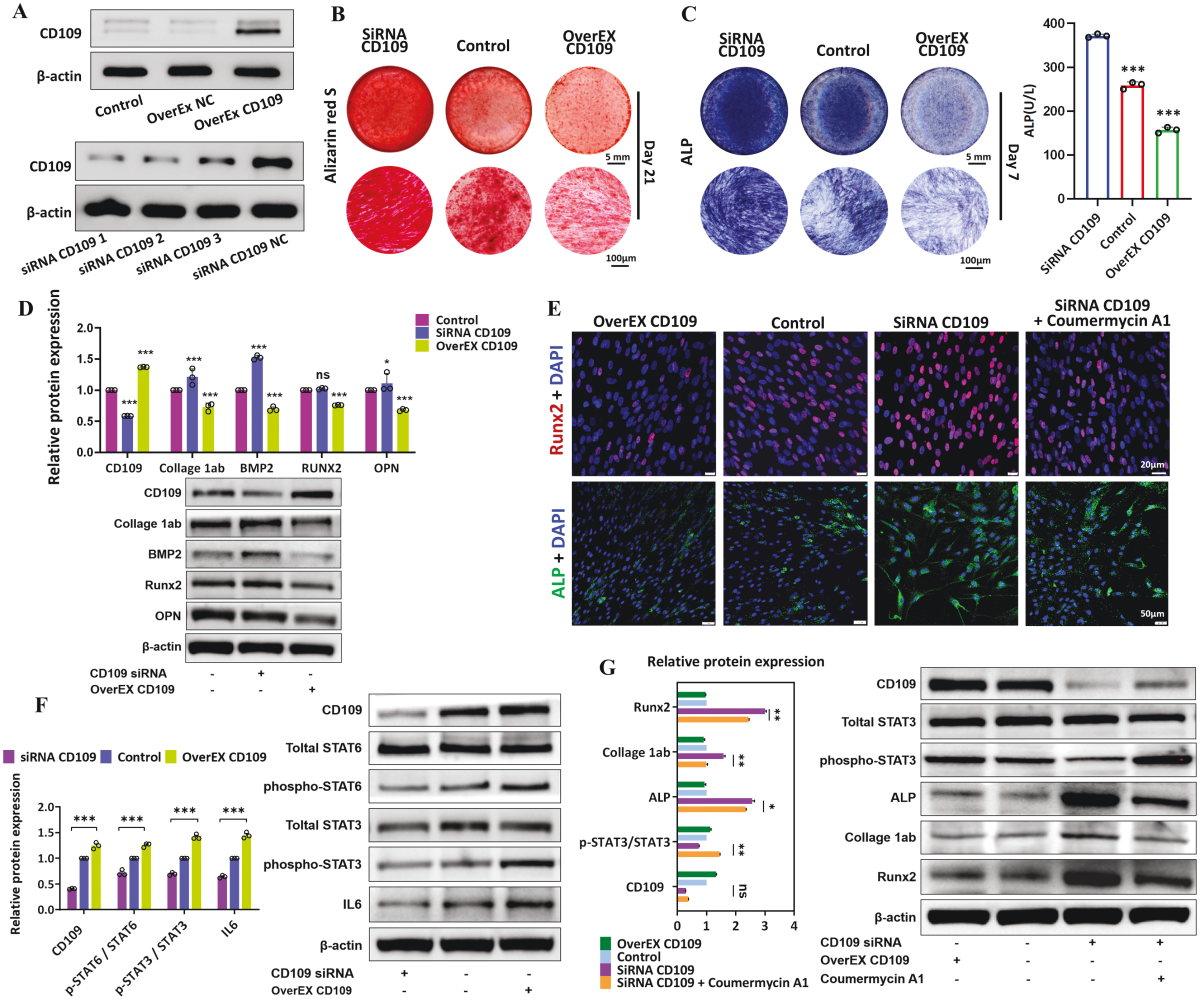
CD109 restraining the osteogenic ability of PDLSCs through the JAK/STAT3 signaling pathway. (A) Representative Western blots results of CD109 expression on hPDLSCs after transfections. *N* ≥ 3. (B) Representative Alizarin red S staining images of hPDLSCs on day 21. *N* ≥ 3. (C) Quantitative analyses of ALP activity and representative ALP staining images of hPDLSCs on day 7. *N* ≥ 3. (D) Western blot results and semi-quantifications of CD109, Collage 1ab, BMP2, Runx2, OPN protein levels on hPDLSCs after transfections with CD109 siRNA and CD109 overexpression plasmids. (E) Representative confocal immunofluorescence images of the expression of Runx2 and ALP in hPDLSCs. *N* ≥ 3. (F-G) Western blot results and semi-quantifications of STAT3, pSTAT3, pSTAT6, STAT6, IL-6, Runx2, Collage 1ab, and ALP protein levels on hPDLSCs after transfections and application of Coumermycin A1. *N* ≥ 3, *P < .05; **P < .01; ***P < 0.001. Values are means ± SD.

To further investigate the effect of CD109 on the osteogenic function of PDLSCs, we used CD109 siRNA and CD109 cDNA plasmids for transfection to achieve partial silencing and overexpression(OverEx) of the CD109 gene on PDLSCs (Figure 2A). The osteogenic effect was directly evaluated by Alizarin red staining and ALP staining, and the results showed that the osteogenic ability of the silencing group was significantly higher than that of the overexpression group (Figure 2B and C). In addition, Western blotting and qRT-PCR results further proved that the expression level of osteogenic-related proteins in the siRNA CD109 group was significantly higher than that of the overEX CD109 group (Figure 2D and Supplementary Figure S2). Furthermore, we conducted a mechanism analysis to explore the influence of CD109 on the osteogenic capacity of PDLSCs and found that the expression of CD109 on PDLSCs was closely related to the phosphorylation of STAT3 and STAT6 (Figure 2F). As JAK serves as a target within the upstream signaling pathway STAT3/STAT6, we added Coumermycin A1, a JAK agonist, to further verify the effect of CD109 on the osteogenic effect of PDLSCs by Western blotting and immunofluorescence. The results showed that the expression levels of osteogenic-related proteins in the CD109 siRNA + Coumermycin A1 group were lower than that in the CD109 siRNA group (Figure 2E and G), which suggested that CD109 inhibited osteogenesis through the JAK/STAT3 signaling pathway.

### CD109 secreted by PDLSCs induces osteoclast formation and M1 macrophage polarization

**Figure 3.**
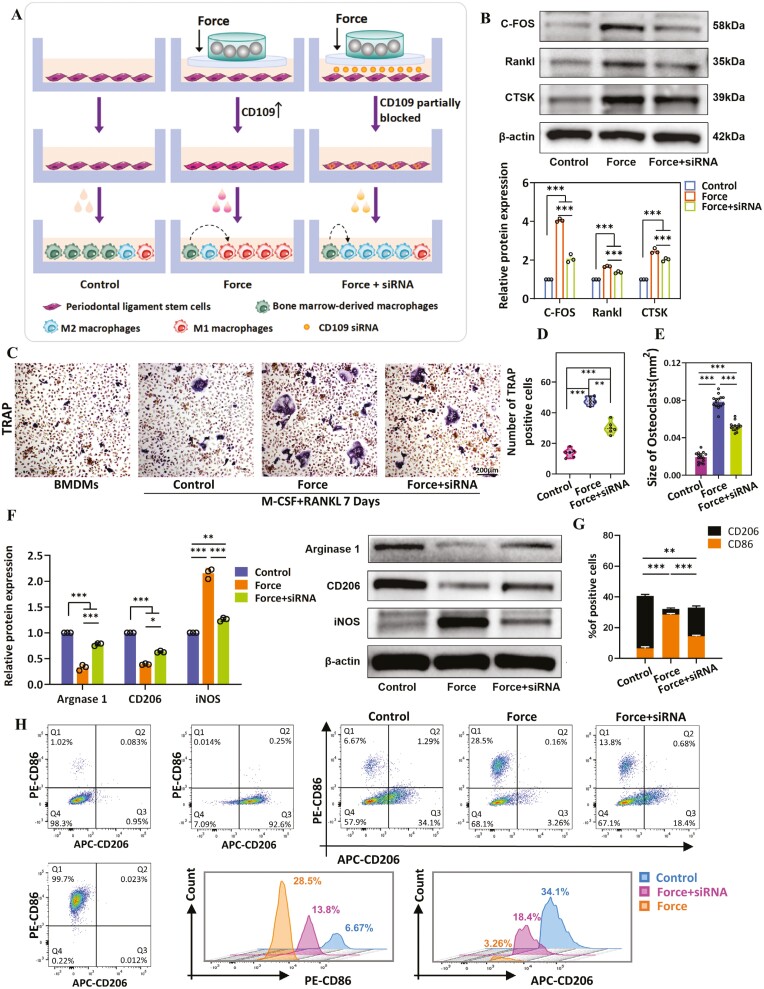
Force-stimulated PDLSCs secreted CD109, promoting the occurrence and development of osteoclasts and M1 macrophage polarization. (A) Schematic illustration of constructing a coculture model of PDLSCs and BMDMs and in vitro therapeutic study. (B) Western blot results and semi-quantifications of C-fos, Rankl, and Ctsk protein expressions in BMDMs on day 7. (C-E) Representative TRAP staining images and quantitative analyses of the size and number of osteoclasts on day 7. *N* ≥ 3. (F) Western blot results and semi-quantifications of arginase-1, CD206, iNOS protein levels on BMDMs. (G-H) Representative FACS analysis of macrophages cultured with conditional medium after 24 hours. *P < .05; **P < .01; ***P < .001. *N* ≥ 3. Values are means ± SD.

To figure out the effect of CD109 expression on force-treated PDLSCs on osteoclast differentiation, we conducted a coculture model of PDLSCs and BMDMs (Figure 3A). In brief, the co-incubated supernatants collected from PDLSCs with or without loading were used to culture BMDMs, along with the soluble receptor activator of Rankl and M-csf to stimulate osteoclast differentiation. The Western blot and qRT-PCR results showed that force treatment of PDLSCs promoted higher levels of C-Fos, Rankl, CTSK, OPG, and Rank in BMDMs (Figure 3B and Supplementary Figure S3), accompanied by a greater number of larger osteoclasts substantially assayed by TRAP staining. By contrast, reducing the CD109 expression on PDLSCs could weaken these impacts (Figure 3C-E). These results indicated that CD109 released by force-stimulated PDLSCs could promote osteoclast differentiation.

What’s more, whether CD109 expression on force-treated PDLSCs could promote BMDMs to polarize toward the M1 phenotype was further explored in this study. To this end, the co-incubated supernatants collected from different pretreatments were applied to rat BMDMs. Western blot analysis and flow cytometry results showed that the expression of M1 markers (iNOS, CD86) were dramatically upregulated in the force group (*P* < .001 vs control) but downregulated in the force + siRNA group (*P* < .001 vs force). Conversely, the protein expression of M2 markers (arginase-1, CD206) were downregulated in the force group (*P* < .001 vs control) but upregulated in the force + siRNA group (*P* < .001 vs force group) (Figure 3F-H). These findings suggested that upregulated CD109 production and elimination by mechanical load-stimulated PDLSCs could promote BMDMs to polarize into the M1 phenotype.

### Inhibition of CD109 suppresses mechanical load-induced alveolar bone remodeling and tooth movement

**Figure 4.**
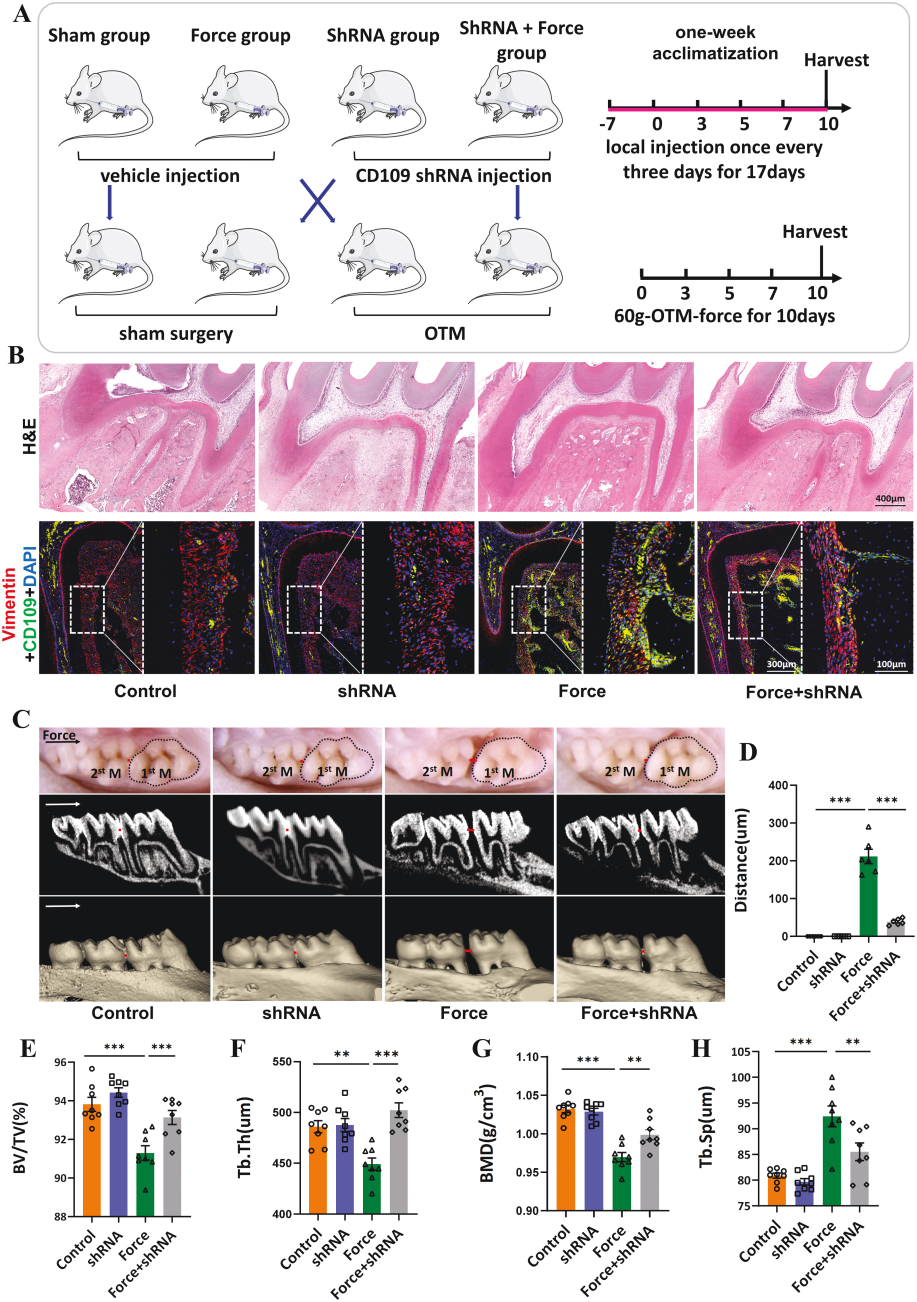
Inhibition of CD109 in PDL represses mechanical load-induced alveolar bone remodeling and tooth movement. (A) Schematic illustration of mechanical force-induced tooth movement SD rat model and in vivo therapeutic study. (B) Representative H&E and Immunofluorescence staining of CD109 and Vimentin in the compression side of roots. *N* = 8. (C) Representative occlusal view and micro-CT images of tooth movement for 10 days in rats. 1st M, the first molar; 2nd M, the second molar. The arrow indicates the direction of mechanical force. The line segment represents the tooth movement distance. (D) Semi-quantitative analysis showed that the mechanical load increased the tooth movement distance. *N* = 8. (E-H) The ratio of trabecular bone volume fraction (BV/TV), trabecular thickness (Tb.Th), trabecular separation (Tb.Sp), and bone mineral density (BMD) in the pressure side alveolar bone of the mesial root of the maxillary first molar was quantified by micro-CT images. *N* = 8. **P < 0.01; *** *P* < 0.001. Values are means ± SD.

To further elucidate the functional significance of CD109 in OTM, we silenced the expression of CD109 in PDL by transfecting lentivirus (Figure 4A and B). After transfection, we used the fluorescence titer detection method to detect the virus titer (Supplementary Figure S4A). Among the 3 LV-shRNAs, LV-sh-CD109-3 exhibited the highest silencing efficiency verified by Western blots (Supplementary Figure S4B and C) and thus was used in the following experiments. The results indicated that following 10 days of OTM, the force group exhibited an increase in tooth movement distance to 220 μm, whereas the shRNA + force group demonstrated a significant reduction in tooth movement distance (54 μm) (*P* < .05; Figure 4C and D).

Micro-CT analysis revealed a significant decrease in BV/TV, Tb.Th, and BMD in the maxillae of force group, while the figures demonstrated partial recovery in the force + shRNA group (Figure 4E-4G). Tb.sp results showed an increase in the degree of bone trabeculae separation during tooth movement. However, the addition of shRNA weakened this increase, although it remained significantly higher than the control group (Figure 4H). These results suggested that mechanical force-induced rats exhibited a bone phenotype resembling osteoporosis with decreased BV/TV, Tb.Th, and BMD, contributing to tooth movement initiation. However, decreasing CD109 expression in PDL when loaded by force could inhibit bone turnover, leading to tooth movement deceleration.

**Figure 5.**
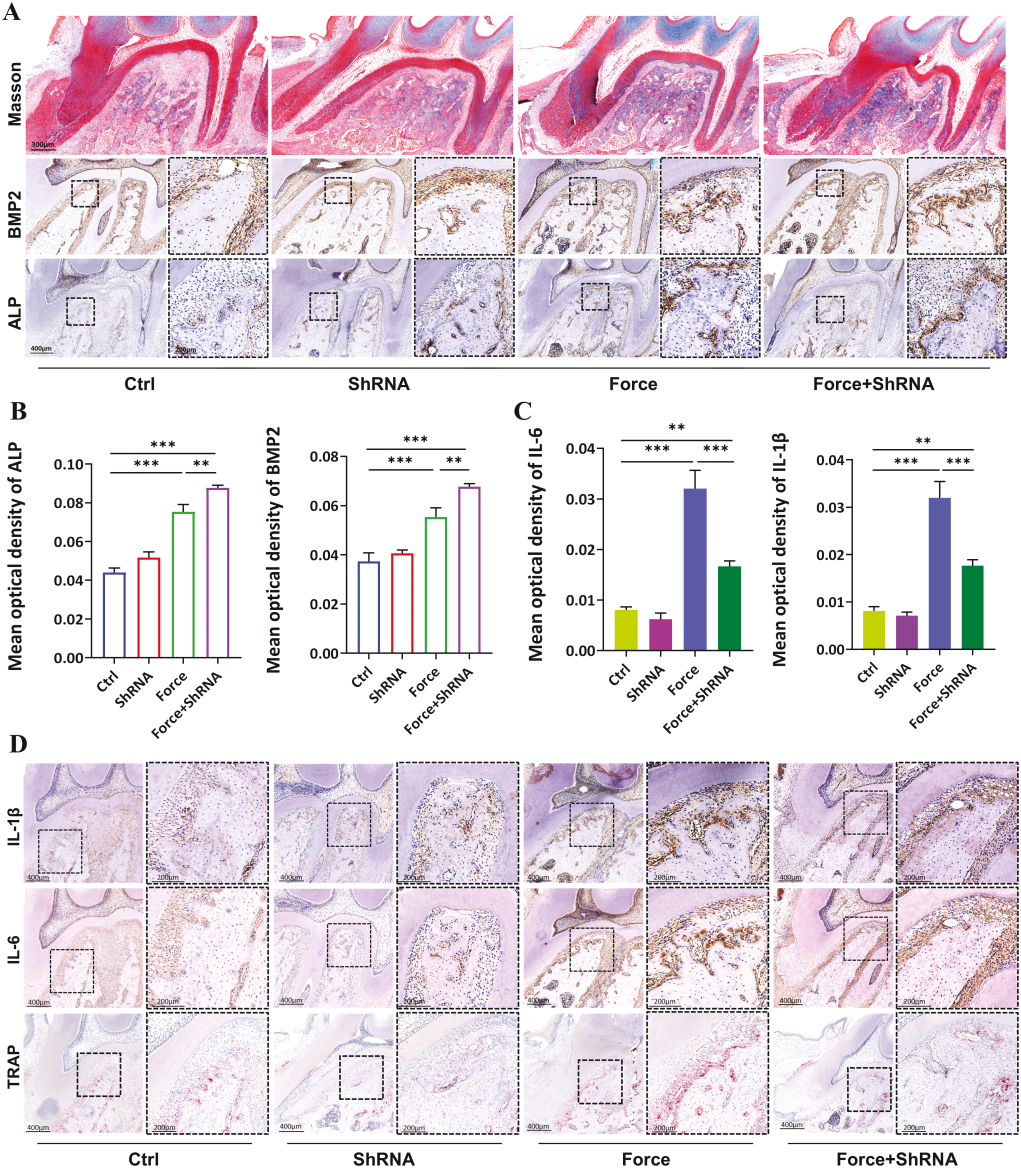
CD109 modulates the in vivo secretion of pro-inflammatory cytokines and influences the osteogenic and osteoclastic potential of PDL. (A) Upper panels show representative Masson staining images of the sagittal cross section of left maxillary first molar of SD rat, and the lower panels show immunohistochemical staining of Alp and Bmp2 on the compressive sides. *N* = 8. (B-C) Semi-quantification of the immunohistochemical staining of Alp, Bmp2, IL-6, and L-1β on the compressive sides of left maxillary first molar of SD rats. *N* = 8. *P < .05; **P < .01; ***P < .001. (means ± SD). (D) The immunohistochemical staining of IL-6 and IL-1β and trap staining of a sagittal cross section of the left maxillary first molar of SD rats. Large boxed areas show high magnification views of the small boxed areas. *N* = 8.

In the process of bone remodeling, mechanical stimulation or bone resorption can effectively promote the osteogenic differentiation of PDLSCs. Masson staining results illustrated a notable increase in new bone mass within the force group, while exhibiting a significant further augmentation in the force + shRNA group (Figure 5A). In addition, immunohistochemical analysis revealed that the expression of ALP and BMP2 in the PDL and adjacent alveolar bone showed an upward trend in the force group, whereas these indexes were significantly increased further in the force + shRNA group (Figure 5A and B). As to osteoclastic regulation, TRAP staining exhibited that the injection of LV-CD109-shRNA reduced the force-enhanced TRAP-positive osteoclasts in the compressive side of PDL and alveolar bone (Figure 5D). Plenty of research has demonstrated the close interaction between inflammatory response and osteoclasts.^19-21^ In our study, the immunohistochemical results showed that the expression of pro-inflammatory factors in the periodontal membrane and adjacent alveolar bone increased under mechanical force while adding LV-CD109-shRNA reduced these affections (Figure 5C and D). These findings indicated that CD109 played an important part in force-induced inflammatory bone remodeling.

### Mechanical force enhances CD109 expression via the repression of miR-340-5p

**Figure 6.**
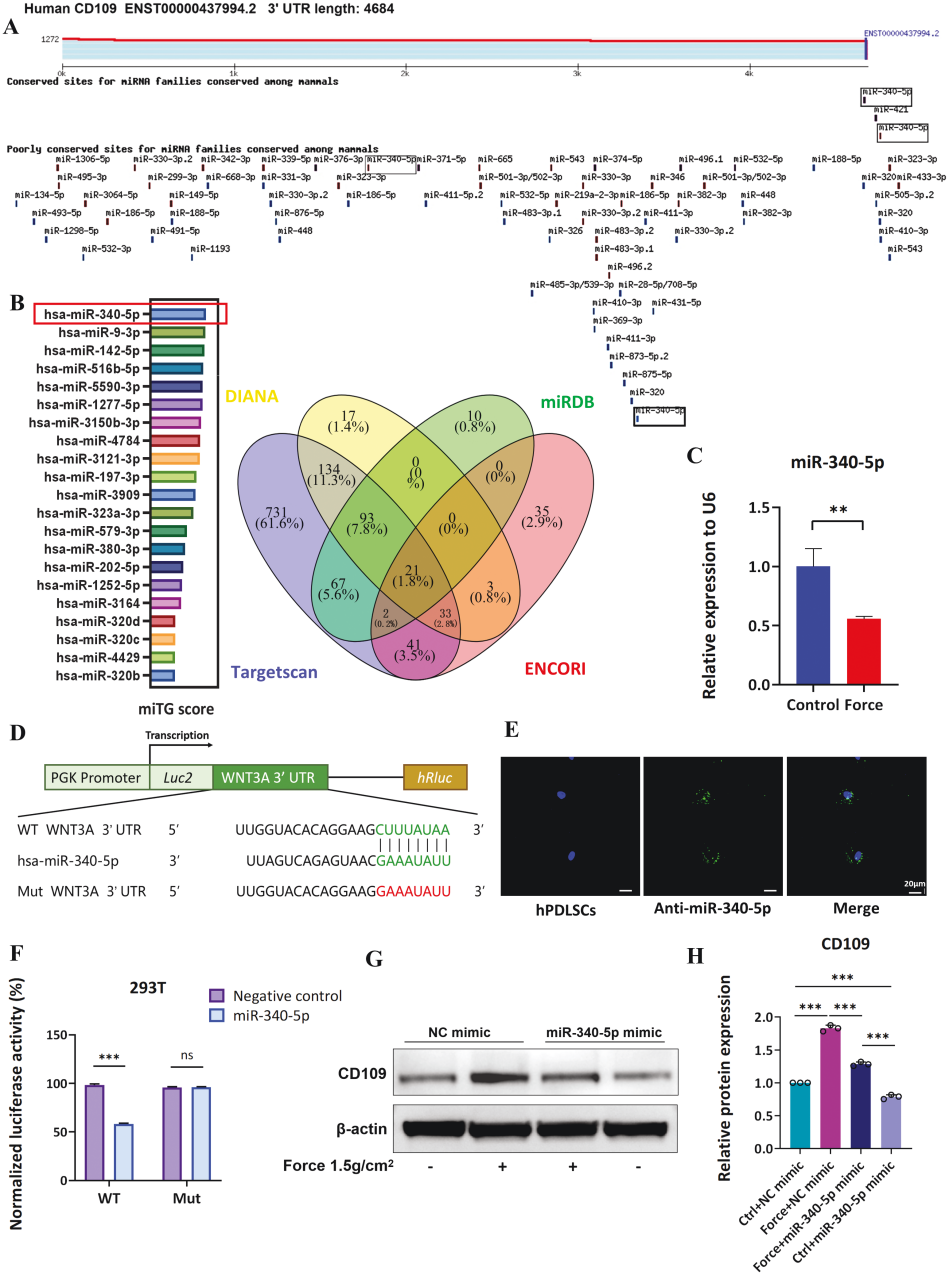
Mechanical force facilitates CD109 expression on PDLSCs via the repression of miR-340-5p. (A) miR-340-5p was predicted as the target miRNA of the human CD109 gene by Targetscan. (B) The Venn diagram shows the targeted miRNAs of the human CD109 gene predicted by the DIANA, miRDB, TargetScan, and ENCORI databases. A total of 21 miRNA with crossover were obtained. (C) Representative miR-340-5p expression on hPDLSCs relative to U6 by qRT-PCR. *N* ≥ 3. (D) Binding sites of miR-340-5p and CD109. Sequence of the binding site, wild-type (WT), and mutant (MUT) sites. (E) Co-localization of hPDLSCs and miR-340-5p. N ≥3. (F) Dual-Luciferase activity assay results of the interaction between miR-340-5p and the 3ʹ-UTR of CD109 on 293T cells. (G-H) Western blot and semi-quantification assessed the levels of CD109 protein on hPDLSCs after transfections and application of loading compressive force. *N* ≥ 3, *P < .05; **P < .01; ***P < .001. Values are means ± SD.

We performed target miRNA prediction for the human CD109 gene, utilizing the Targetscan, DIANA, miRDB, and ENCORI databases and obtaining their intersection (Figure 6A). Ultimately, we retrieved the results by calculating the miTG score (Figure 6B). After that, we conducted qRT-PCR analysis on the top 3 miRNAs based on their scores, revealing a significant downregulation of miR-340-5p expression in the Force group compared to the control group (Figure 6C and Supplementary Figure S5). The MicroRNA sequences are listed in Supplementary Figure Table S4. The luciferase reporter assay results revealed the presence of an interaction between CD109 and hsa-miR-340-5p, as demonstrated in our study (Figure 6D and F). To further investigate whether miR-340-5p is involved in the process of mechanical force-induced CD109 expression, Fluorescence-labeled miR-340-5p mimic was transfected into hPDLSCs, and the co-localization of the miR-340-5p mimic and hPDLSCs were detected by microscopy (Figure 6E). Western blot results showed that the expression of CD109 in the force + miR-340-5p mimic group was lower than that in the force + NC mimic group (Figure 6G and H), indicating that miR-340-5p inhibited mechanical force-mediated CD109 activation on PDLSCs.

## Discussion

Translating mechanical stimuli into biochemical responses is crucial for intercellular communication in the osseous tissue, which potentially regulates the immune microenvironment and bone remodeling in mammals.^22,23^ In this process, MSCs that are exposed to mechanical force play a vital part in reaction and regulation, involving various cytokines and signaling pathways.^3,24,25^ However, the key molecules and underlying mechanisms require further exploration. To this end, we established an animal model of force-induced tooth movement and an in vitro experiment with continuous compression. OTM is caused by a directional and appropriate mechanical force that triggers a sterile inflammatory response in the periodontal tissues, followed by a coordinated process of bone resorption on the pressure side and bone formation on the tension side.^1,2^ Meaningfully, our study demonstrated a CD109-mediated mechanical force response machinery on PDLSCs in governing osteogenesis and osteoclast, indicating a novel target of mechanical signal transduction for MSCs to regulate immune response and bone remodeling. This suggests that it is necessary to regulate the CD109 expression through controlling the force during orthodontic clinical treatment, especially when treating adult patients and patients with periodontitis, who show a deterioration in the regenerative capacity of periodontal tissues. This approach may help these patients maintain the balance of alveolar bone remodeling and prevent tooth loosening during OTM.

CD109, a glycoprotein expressed on the surface of various cell types, has demonstrated the association with the occurrence and progression of diverse diseases, establishing it as a validated therapeutic target applied in clinic.^26-28^ However, few studies reported the alteration of CD109 expression on MSCs under mechanical stimuli and the corresponding functions. Notably, several studies have revealed the role of CD109 in bone immune regulation and metabolic disorders, but there remains controversy. An evaluation of 4 male CD109 knockout mice indicated that a deficiency in CD109 may lead to a high-turnover phenotype resembling osteoporosis.^12^ On the contrary, as per the International Mouse Phenotyping Consortium (IMPC, www.mousephenotype.org), an examination of 7 male CD109 knockout mice revealed a significant elevation in bone mineral density (*P* = 9 × 10^–4^) and bone mineral content (*P* = 3.3 × 10^–4^).^29^ These findings underscored the diverse impact of CD109 on bone physiology in mice, which could be influenced by distinct substrains of mice exhibiting varying levels and manifestations of mutational effects.^30^ In our study, we have successfully demonstrated that the compressive mechanical stimulation upregulated CD109 expression on PDLSCs through both in vivo and in vitro experiments. Inhibition of CD109 expression resulted in an increase in bone density and a reduction of tooth movement distance. These results suggest a novel role of CD109 as a mechanical transducer of PDLSCs in activating alveolar bone turnover.

CD109 acts as a negative receptor of the TGF-βsignaling pathway in multiple cell types.^10^ However, several recent studies revealed CD109 mediates certain pathological processes through other mechanisms, like a glycolytic pathway,

JNK/MAPK signaling pathway and STAT3-NOTCH-1 signaling axis.^14,31,32^ In this study, we confirmed that the JAK/STAT3 signaling pathway might contribute to CD109-mediated repression of PDLSCs osteogenic differentiation, providing a novel insight into the mechanisms of CD109 in regulating MSC behaviors. What’s more, CD109 has also been implicated in the development of osteoclasts.^13^ Knocking out the CD109 gene in RAW264.7 cells indicated that monocytes lacking CD109 expression were less likely to undergo fusion and form large multinucleated osteoclasts, suggesting CD109 might serve as a crucial regulator of osteoclast formation.^13^ Several studies showed that CD109 could be released into the extracellular space and played a significant role as a soluble protein in various diseases.^33-36^ However, there is a lack of research to investigate the impact of secreted CD109 on osteoclastogenesis. In our study, the culture supernatant of PDLSCs with higher expression of CD109 promoted both the number and volume of multinucleated osteoclasts, indicating PDLSCs induced the formation and differentiation of osteoclasts through paracrine secretion of CD109. These findings uncover a novel insight into the potential of CD109 as a therapeutic target in bone metabolic diseases.

In addition to osteoclast, MSCs and their released extracellular vesicles (EVs) possess the potential to influence the functions of most immune effector cells.^37^ In our study, we discovered that PDLSCs exerted a positive regulatory effect on the polarization of macrophages toward the M1 phenotype through paracrine secretion of CD109. Furthermore, as IL-6 and IL-1β are the key inflammatory mediators within M1 macrophages, we observed a decrease in the expression of IL-6 and IL-1β in the periodontal ligament when CD109 was silenced in vivo. These findings suggest that CD109 released by PDLSCs may attenuate immune-inflammatory responses in the local microenvironment, validating CD109 as an effector of PDLSCs in immunomodulation. Moreover, CD109 has been identified to be secreted through localizing on EVs,^36^ providing a new strategy for CD109 in regulating the immune microenvironment.

MiRNAs are a subgroup of non-coding RNAs with 19-22 nucleotides in length, which degrade mRNA of a targeted gene through pairing with specific regions of 3ʹUTR.^38^ During bone remodeling, miRNAs target or indirectly mediate osteogenic or osteoclastogenic signaling factors and downstream cascades.^39,40^ Particularly, miRNAs are mechanosensitive and can convert mechanical signals through integrins, cytoskeleton, and ion channels.^41^ For instance, the mechanical load could decrease the expression of miR-325-3p to facilitate chondrocyte senescence.^42^ Additionally, miR-140 could target RhoA to transduce mechanical signal into biological pathways, suppressing osteogenic differentiation of PDLSCs.^43^ Our investigation revealed that by directly targeting CD109, miR-340-5p reduced by mechanical force could elevate the CD109 expression on PDLSCs, consequently leading to the activation of bone remodeling. These findings provide compelling evidence of miR-340-5 as a mechanical sensor in PDLSC-mediated alveolar bone remodeling.

## Conclusions

**Figure 7.**
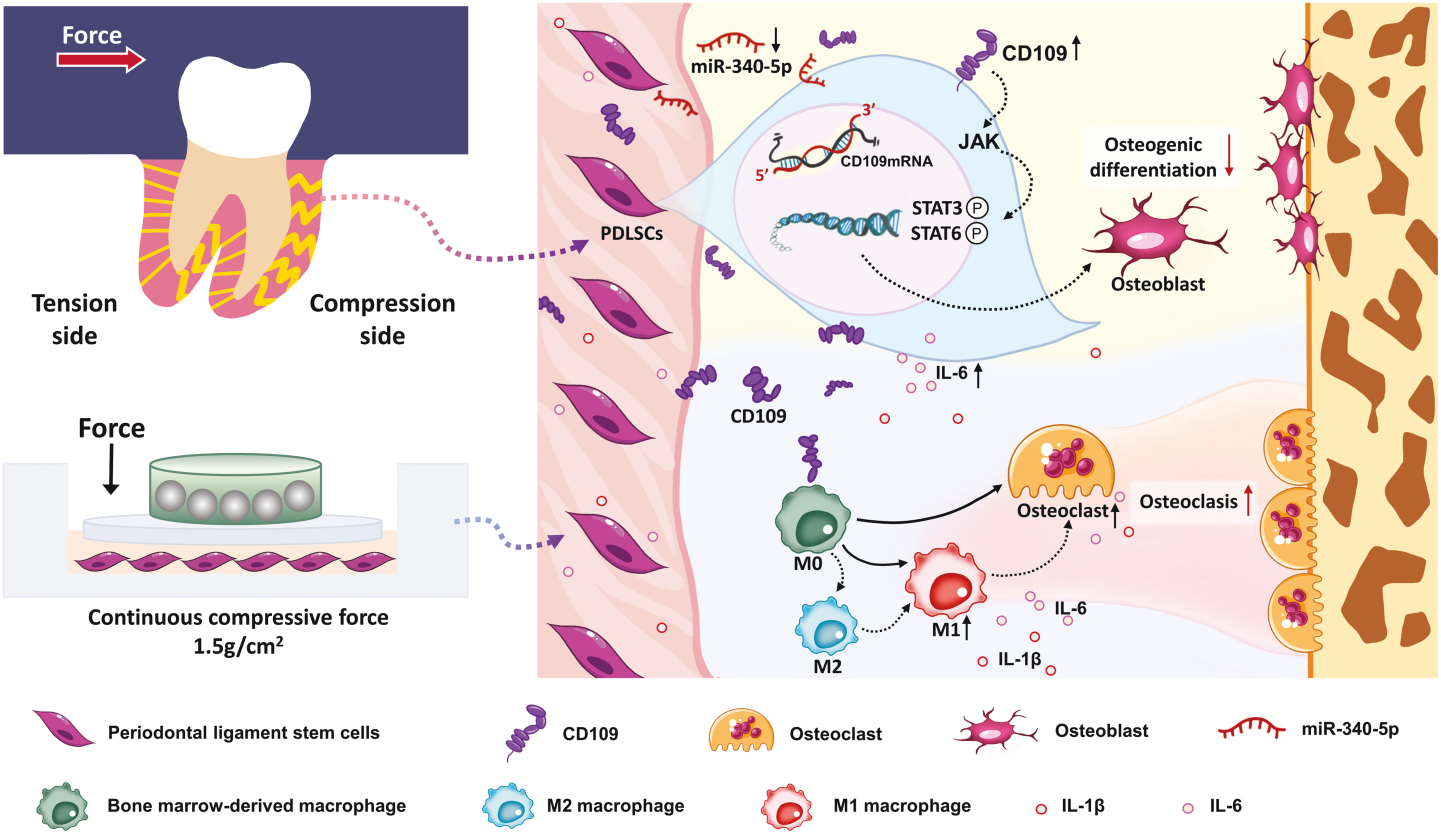
Schematic illustration of how CD109 as a mechanical transducer regulates immune microenvironment and bone remodeling during OTM.

Our study indicates that mechanical load-activated CD109 expressed on PDLSCs restrains the osteogenesis capacity of PDLSCs through the JAK/STAT3 signaling pathway. It can also be released to promote PDLSC-induced osteoclast formation and M1 macrophage polarization. This CD109-mediated mechanical force response machinery in PDLSCs depends on the repression of miR-340-5p, which contributes to regulating inflammatory alveolar bone remodeling (Figure 7). These findings provide novel insights into the mechanisms of force-involved bone remodeling and the therapeutic strategies for bone metabolic diseases.

## Supplementary material

Supplementary material is available at *Stem Cells Translational Medicine* online.

szae035_suppl_Supplementary_Material

## Data Availability

The data that support the findings of this study are available from the corresponding author upon reasonable request.
