# Supplementary material for: Mechanical force-activated CD109 on periodontal ligament stem cells governs osteogenesis and osteoclast to promote alveolar bone remodeling
Source: Stem Cells Transl Med. 2024 Jun 17;13(8):812–25. doi: 10.1093/stcltm/szae035 (PMC11328932; doi:10.1093/stcltm/szae035)
Supplement: szae035_suppl_Supplementary_Material [file szae035_suppl_supplementary_material.docx]

***Supplementary Material for***

**Mechanical force-activated CD109 on periodontal ligament stem cells governs osteogenesis and osteoclast to promote alveolar bone remodeling**

Yang Li^a#^, Yi Li^a#^, Chao Liu^a#^, Xinyi Yu^a^, Ziqi Gan^a^, Lusai Xiang^a^, Jinxuan Zheng^a^, Bowen Meng^a^, Rongcheng Yu^a^, Xin Chen^a^, Xiaoxing Kou^a,b,^*, Yang Cao^a,^*, Tingting Ai^a,^*

*^a^ Hospital of Stomatology, Guangdong Provincial Key Laboratory of Stomatology, Guanghua School of Stomatology, Sun Yat-sen University, Guangzhou 510055, China*

*^b^ South China Center of Craniofacial Stem Cell Research, Hospital of Stomatology, Sun Yat-sen University, 74 Zhongshan 2Rd, Guangzhou 510080, China*

* Corresponding author:

Email: aitt3@mail.sysu.edu.cn (T, Ai); [caoyang@mail.sysu.edu.cn](mailto:caoyang@mail.sysu.edu.cn) (Y, Cao); kouxiaoxing@mail.sysu.edu.cn (X, Kou)

^#^Y. Li, Y. Li and C. Liu contributed equally to this study.

**Corresponding author**

**Tingting Ai, DDS, PhD**

Hospital of Stomatology, Guangdong Provincial Key Laboratory of Stomatology, Guanghua School of Stomatology, Sun Yat-sen University

Dept. of Orthodontics

Lingyuan West Road 56

Guangzhou, Guangdong 510055, China

(Tel) + 86-20-83802802.

mail: aitt3@mail.sysu.edu.cn

**1 Supplementary tables**

**Table S1. siRNA for gene silencing**

| **siRNA duplex** | Reverse primer (5ʹ-3ʹ) |
| --- | --- |
| genOFFTM st-h-CD109_001 | GCAACCAACTGACTCTTGA |
| genOFFTM st-h-CD109_002 | GCAACATGATTACATCATT |
| genOFFTM st-h-CD109_003 | GGGAGAAATTCCTATCACA |

**Table S2. cDNA ORF sequences for gene overexpression**

| **Human CD109 cDNA ORF clone** |
| --- |
| atgcagggcccaccgctcctgaccgccgcccacctcctctgcgtgtgcaccgccgcgctggccgtggctcccgggcctcggtttctggtgacagccccagggatcatcaggcccggaggaaatgtgactattggggtggagcttctggaacactgcccttcacaggtgactgtgaaggcggagctgctcaagacagcatcaaacctcactgtctctgtcctggaagcagaaggagtctttgaaaaaggctcttttaagacacttactcttccatcactacctctgaacagtgcagatgagatttatgagctacgtgtaaccggacgtacccaggatgagattttattctctaatagtacccgcttatcatttgagaccaagagaatatctgtcttcattcaaacagacaaggccttatacaagccaaagcaagaagtgaagtttcgcattgttacactcttctcagattttaagccttacaaaacctctttaaacattctcattaaggaccccaaatcaaatttgatccaacagtggttgtcacaacaaagtgatcttggagtcatttccaaaacttttcagctatcttcccatccaatacttggtgactggtctattcaagttcaagtgaatgaccagacatactatcaatcatttcaggtttcagaatatgtattaccaaaatttgaagtgactttgcagacaccattatattgttctatgaattctaagcatttaaatggtaccatcacggcaaagtatacatatgggaagccagtgaaaggagacgtaacgcttacatttttacctttatccttttggggaaagaagaaaaatattacaaaaacatttaagataaatggatctgcaaacttctcttttaatgatgaagagatgaaaaatgtaatggattcttcaaatggactttctgaatacctggatctatcttcccctggaccagtagaaattttaaccacagtgacagaatcagttacaggtatttcaagaaatgtaagcactaatgtgttcttcaagcaacatgattacatcattgagttttttgattatactactgtcttgaagccatctctcaacttcacagccactgtgaaggtaactcgtgctgatggcaaccaactgactcttgaagaaagaagaaataatgtagtcataacagtgacacagagaaactatactgagtactggagcggatctaacagtggaaatcagaaaatggaagctgttcagaaaataaattatactgtcccccaaagtggaacttttaagattgaattcccaatcctggaggattccagtgagctacagttgaaggcctatttccttggtagtaaaagtagcatggcagttcatagtctgtttaagtctcctagtaagacatacatccaactaaaaacaagagatgaaaatataaaggtgggatcgccttttgagttggtggttagtggcaacaaacgattgaaggagttaagctatatggtagtatccaggggacagttggtggctgtaggaaaacaaaattcaacaatgttctctttaacaccagaaaattcttggactccaaaagcctgtgtaattgtgtattatattgaagatgatggggaaattataagtgatgttctaaaaattcctgttcagcttgtttttaaaaataagataaagctatattggagtaaagtgaaagctgaaccatctgagaaagtctctcttaggatctctgtgacacagcctgactccatagttgggattgtagctgttgacaaaagtgtgaatctgatgaatgcctctaatgatattacaatggaaaatgtggtccatgagttggaactttataacacaggatattatttaggcatgttcatgaattcttttgcagtctttcaggaatgtggactctgggtattgacagatgcaaacctcacgaaggattatattgatggtgtttatgacaatgcagaatatgctgagaggtttatggaggaaaatgaaggacatattgtagatattcatgacttttctttgggtagcagtccacatgtccgaaagcattttccagagacttggatttggctagacaccaacatgggttacaggatttaccaagaatttgaagtaactgtacctgattctatcacttcttgggtggctactggttttgtgatctctgaggacctgggtcttggactaacaactactccagtggagctccaagccttccaaccatttttcatttttttgaatcttccctactctgttatcagaggtgaagaatttgctttggaaataactatattcaattatttgaaagatgccactgaggttaaggtaatcattgagaaaagtgacaaatttgatattctaatgacttcaaatgaaataaatgccacaggccaccagcagacccttctggttcccagtgaggatggggcaactgttctttttcccatcaggccaacacatctgggagaaattcctatcacagtcacagctctttcacccactgcttctgatgctgtcacccagatgattttagtaaaggctgaaggaatagaaaaatcatattcacaatccatcttattagacttgactgacaataggctacagagtaccctgaaaactttgagtttctcatttcctcctaatacagtgactggcagtgaaagagttcagatcactgcaattggagatgttcttggtccttccatcaatggcttagcctcattgattcggatgccttatggctgtggtgaacagaacatgataaattttgctccaaatatttacattttggattatctgactaaaaagaaacaactgacagataatttgaaagaaaaagctctttcatttatgaggcaaggttaccagagagaacttctctatcagagggaagatggctctttcagtgcttttgggaattatgacccttctgggagcacttggttgtcagcttttgttttaagatgtttccttgaagccgatccttacatagatattgatcagaatgtgttacacagaacatacacttggcttaaaggacatcagaaatccaacggtgaattttgggatccaggaagagtgattcatagtgagcttcaaggtggcaataaaagtccagtaacacttacagcctatattgtaacttctctcctgggatatagaaagtatcagcctaacattgatgtgcaagagtctatccattttttggagtctgaattcagtagaggaatttcagacaattatactctagcccttataacttatgcattgtcatcagtggggagtcctaaagcgaaggaagctttgaatatgctgacttggagagcagaacaagaaggtggcatgcaattctgggtgtcatcagagtccaaactttctgactcctggcagccacgctccctggatattgaagttgcagcctatgcactgctctcacacttcttacaatttcagacttctgagggaatcccaattatgaggtggctaagcaggcaaagaaatagcttgggtggttttgcatctactcaggataccactgtggctttaaaggctctgtctgaatttgcagccctaatgaatacagaaaggacaaatatccaagtgaccgtgacggggcctagctcaccaagtcctgtaaagtttctgattgacacacacaaccgcttactccttcagacagcagagcttgctgtggtacagccaacggcagttaatatttccgcaaatggttttggatttgctatttgtcagctcaatgttgtatataatgtgaaggcttctgggtcttctagaagacgaagatctatccaaaatcaagaagcctttgatttagatgttgctgtaaaagaaaataaagatgatctcaatcatgtggatttgaatgtgtgtacaagcttttcgggcccgggtaggagtggcatggctcttatggaagttaacctattaagtggctttatggtgccttcagaagcaatttctctgagcgagacagtgaagaaagtggaatatgatcatggaaaactcaacctctatttagattctgtaaatgaaacccagttttgtgttaatattcctgctgtgagaaactttaaagtttcaaatacccaagatgcttcagtgtccatagtggattactatgagccaaggagacaggcTgtgagaagttacaactctgaagtgaagctgtcctcctgtgacctttgcagtgatgtccagggctgccgtccttgtgaggatggagcttcaggctcccatcatcactcttcagtcatttttattttctgtttcaagcttctgtactttatggaactttggctg |

**Table S3. The qPCR primer sequences for target genes**

| **Gene** | **Forward primer (5ʹ-3ʹ)** | **Reverse primer (5ʹ-3ʹ)** |
| --- | --- | --- |
| *Human CD109* | TTATGAGGTGGCTAAGCAGGC | CTGTACCACAGCAAGAGGACTT |
| *Human IL-1β* | ATGATGGCTTATTACAGTGGCAA | GTCGGAGATTCGTAGCTGGA |
| *Human TNF-α* | CCCATCTATCTGGGAGGGGT | GCGTTTGGGAAGGTTGGATG |
| *Human ALP* | GCCCTCTCCAAGACATATA | CCATGATCACGTCGATATCC |
| *Human RUNX2* | GCACAAACATGGCCAGATTCA | AAGCCATGGTGCCCGTTAG |
| *Human β-ACTIN* | CACCATTGGCAATGAGCGGTTC | AGGTCTTTGCGGATGTCCACGT |
| Rat Opg | GACCAAAGTGAATGCCGAGAG | CGCTGCTTTCACAGAGGTCAA |
| Rat Cathepsin K | CGGCTATATGACCACTGCCTTC | TTTGCCGTGGCGTTATACATACA |
| Rat Rankl | CTCATGCAGGAGAATCAAAC | TTCCATCATAGCTGGAACTC |
| Rat Rank | CAGGACAGGGCTGATGCAA | TGACTGACGTACACCACGATGA |
| Rat C-fos | CGTCTTCCTTTGTCTTCACCTACC | TTGCTGCTGCTGCCCTTT |
| Rat β-actin | AFAFFFAAATCGTGCGTGAC | GGCCGTCAGGCAGCTCATAG |

**Table S4. MicroRNA sequences**

| **miRNA name** | **miRBase Accession** | **Mature sequeance** |
| --- | --- | --- |
| hsa-miR-142-5p | MIMAT0000433 | CAUAAAGUAGAAAGCACUACU |
| hsa-miR-340-5p | MIMAT0004692 | UUAUAAAGCAAUGAGACUGAUU |
| hsa-miR-9-3p | MIMAT0000442 | AUAAAGCUAGAUAACCGAAAGU |

**2 Supplementary methods**

***Construction of rCd109-shRNA***

### To silence CD109, three small hairpin RNAs (shRNA) were inserted into U6-MCS-CMV-zsGreen-PGK-Puromycin lentiviral RNAi vector. The shRNA targeting sequences (5ʹ-3ʹ) were rCd109-shRNA-1: GCACAAGGCAAACAAGATTT ; rCd109-shRNA-2: GGTTAACATTTGACAGCAAGA and rCd109-shRNA-1: GCAAACCTTGTAAGAGATAAC, and empty lentivectors were used as negative control (NC).

***Packaging, concentration, and purification of lentiviruses***

Inoculate 293T cells into a 10 cm cell culture dish and transfect them with the vector plasmid PFV:PLVX, along with the helper plasmids psPAX2 (pHelper 1) and pMD2G (pHelper2), when the cell density reaches 70% to 80%. After transfection for 24 hours, observe the number of cells containing labeled fluorescence (GFP / RFP) under a microscope to determine successful transfection (fluorescent cell ratio ≥ 70%), and perform two rounds of supernatant collection. To concentrate and purify lentiviral particles, filter the harvested medium containing viral particles through a 0.22 μm membrane and centrifuge at high speed (80,000 g, 4 h); discard the supernatant, resuspend the pellet in Virus store buffer, and filter sterilize it again through a 0.22 μm membrane.

***Fluorescence-based titration for the detection of lentivirus quality.***

### Inoculate 1 × 10^4^ cells per well of a 96-well plate with 293T cells. Transfect the cells with virus using volumes of 10 μl, 1 μl, 10^-1^ μl, and 10^-2^ μl. After incubating for 72 hours, observe the number of cells containing the target virus marker (GFP or RFP) under a microscope and count the viral titer.

***In vivo validation of CD109 silencing efficiency***

CD109 silenced models were created by transfecting lentivirus into the periodontal ligament (PDL) of SD rats. LV-shRNA CD109 was injected into three specific sites in the PDL of the left maxillary first molars in rats. Each site received a 30ul injection of LV-shRNA CD109 (18 × 10^6^ TU) every three days. After 10 days of feeding, the periodontal tissues of the rats were collected for western blot detection to assess the efficiency of silencing. The LV-shRNA with the highest silencing efficiency was selected for further experiments.

***Culture of human PDLSCs***

The SUN-YAT-SEN University Ethical Committee approved the Isolation of human primary culture PDLSCs protocols, and informed consents were signed by the patients (KQEC-2024-19-01). Briefly, the PDL scraped from the premolar root surface was digested in a mixture of 4 mg/ml typeⅠcollagenase and 6 mg / ml dispase for 15 min at 37°C. The cell culture was initiated by utilizing the single-cell suspensions obtained after filtration of the incubated mixture through a 70-μm strainer. The hPDLSCs were cultured in T25 cell culture flask using α-MEM supplemented with 10% fetal bovine serum (FBS), 100 μg / ml streptomycin and 100 U / ml penicillin (PS). The cells were maintained at 37℃ in a humidified atmosphere containing 5% CO_2_, with the culture medium being refreshed every three days. Before use, the third passage hPDLSCs underwent identification procedures using flow cytometry to indicate the expression pattern of mesenchymal stem cell markers CD105, CD146, STRO1, CD90 and hematopoietic marker CD45. The hPDLSCs were utilized between the third and fifth passages. Coumermycin A1(10 μM, MCE) was applied to activate the expression of JAK, dimethyl sulfoxide (DMSO) was used as control.

***Immunohistochemical staining***

Immunohistochemical staining was performed as previously described^16^. The slices were stained with antibodies including anti-ALP (1:200, 381009, Zenbio), anti-BMP2 (1:100, AF5163, Affinity), anti-IL-6 (1:200, GB11117, Servicebio) and anti-IL-1β(1:200; GB11113, Servicebio). Scanning was performed using Aperio AT2 (Leica), resulting in the visualization of brownish-yellow staining indicative of positive expression.

***Immunofluorescence staining***

Immunofluorescence staining was performed as previously described^16^. The slices were double stained with antibodies consisting of anti-CD109 (1:50; sc-271085, Santa Cruz) and anti-Vimentin (1:300; GB11192, Servicebio) to detect the influence of mechanical force on the CD109 expression in the periodontal tissues. In addition, after different treatments with hPDLSCs, the cells were fixed and permeabilized in 4% paraformaldehyde including 0.15% Triton X-100 for about 20 min. After that, the cells were incubated with primary antibodies anti-RUNX2 (1:300; 12556S, Cell Signaling), or anti-BMP2 (1:200, AF5163, Affinity) antibodies for 12–16 h at 4 ◦C after being blocked with goat serum for 1 hour. The cells were labeled with corresponding secondary antibodies and DAPI on the following day. The slices were scanned (3Dhistech, Panoramic, Budapest, Hungary) to obtain high-resolution images, and the cells were taken with a confocal microscope (Olympus FV3000, Japan).

***Tartrate-resistant acid phosphate (TRAP) assay***

After immersing the dewaxed sections in distilled water at 37°C for 2 hours, they were subsequently incubated with TRAP incubation solution (Servicebio) at the same temperature for 30 minutes. Hematoxylin staining was then performed. Dehydration was carried out using xylene, anhydrous alcohol and 75% ethanol. Finally, the sections were sealed with neutral gum. Scanning of the slices was conducted using Aperio AT2 (Leica), and observation and quantification of multinucleated TRAP-positive cells were performed on the pressure side of the upper-left first molars. In vitro, the cells were fixed using a 4% paraformaldehyde solution for 30 minutes after removal of the culture medium. Subsequently, they were exposed to TRAP dye solution (Sigma-Aldrich, Darmstadt, Germany) at a temperature of 37 °C in darkness for half an hour and enumerated under a light microscope (Axio; Zeiss, Oberkochen, Germany).

***Cell viability assay***

Cell viability and proliferation were assessed using a Cell Counting Kit-8 assay (CCK-8, Dojindo). Briefly, PDLSCs were exposed to mechanical compressive forces ranging from 0.5 g / cm^2^ to 2 g / cm^2^ for varying durations of 0 h, 6 h, 12 h, 18 h and 24 h. These cells were then cultured in a standard 96-well plate according to the experimental protocol. After one day, each well's absorbance at 450 nm was determined using a microplate reader (Tecan SUNRISE microplate reader, Switzerland).

***Terminal Deoxynucleotidyl Transferase-Mediated dUTP Nick End Labeling (TUNEL)Assay***

According to the manufacturer's instructions, tunel assay was performed on hPDLSCs using a One-step TUNEL Assay Kit (E-CK-A322, Elabscience, China). The quantification of apoptotic cells was examined under a fluorescence microscope from Olympus.

***Culture of bone-marrow-derived macrophages (BMDMs)***

Primary rBMDMs were extracted from tibiae and femurs of Sprague-Dawley rats. In brief, a Red Blood Cell Lysis Buffer was used to remove red blood cells from the bone marrow first, and the rest bone marrow cells were cultured in a 37°C, 5% CO_2_ sterile incubator for 48 h to separate rat bone marrow monocytes (BMMs) from other adherent cells. Then non-adherent rBMMs were collected and stimulated with 30 ng / mL macrophage colony-stimulating factor (M-CSF) for the next three or four days until over 95% of non-adherent rBMMs became adherent rBMDMs.

***Luciferase reporter assay***

The wild-type (Wt) or mutant (Mut) CD109 3′-UTR fragments were then subcloned into the pGL3 luciferase promoter vector (Promega, USA). 293T cells were co-transfected with the constructs and either miR-340-5p mimic or mimic control using Lipofectamine 3000 (Invitrogen, USA) for 24h. Luciferase activity was assayed using a Dual-Luciferase Reporter Assay Kit (Promega, USA), and using a dual-luciferase assay system (Promega) to detect.

***Flow cytometric analysis***

BMDMs were labeled with CD86 antibody (0.5 µg / test, 12-0860-83, Invitrogen) and CD206 (MMR) antibody (0.06 µg / test, 17-2069-42, Invitrogen) to measure the expression of macrophage phenotypic markers according to the manufacturer's instructions. After incubating with the primary antibodies for 30 minutes at a temperature of 4°C, the cells were washed with fluorescence activating cell sorter (FACS) buffer, resuspended in FACS buffer and analyzed by the BD FACS Calibur system (BD Biosciences, San Diego, CA). Subsequently, the data were analyzed using FlowJo 10.0.

***Western blot analysis***

Western blot tests were performed as previously described^16^. The primary antibodies included anti-β-actin antibody (1:1000, 200068-8F10, Zenbio), anti-CD109 (1:1000,1:50; sc-271085, Santa Cruz), anti-Collage 1ab antibody (1:1000, 72026S, Cell Signaling), anti-BMP2 antibody (1:1000, AF5163, Affinity), anti-RUNX2 antibody (1:1000, 12556S, Cell Signaling), anti-Osteopontin (OPN) antibody (1:1000, AF0227, Affinity), anti-Arginase 1 antibody (1:1000, #93668, Cell Signaling), anti-CD206 antibody (1:1000, JF0953, HUABIO), anti-iNOS antibody (1:1000, 13120S, Cell Signaling), anti-RANKL antibody (1:1000, ab239607, Abcam), anti-Cathepsin K (CTSK) antibody (1:1000, R381730, Zenbio), anti-STAT3 antibody (1:1000, R380907, Zenbio), anti-Phospho-STAT3 (p-STAT3) antibody (1:1000, R25804, Zenbio), anti-STAT6 antibody (1:1000, ab217998, abcam), anti-Phospho-STAT6 (p-STAT6) antibody (1:1000, ab263947, abcam), anti-IL-6 antibody (1:1000, 500286, Zenbio), anti-ALP antibody (1:1000, 381009, Zenbio), The blots were developed with secondary antibodies conjugated to horseradish peroxidase, followed by chemiluminescence detection and photographic enhancement. The grayscale was quantified using ImageJ 1.37v software (Wayne Rasband).

**3 Supplementary figures**

**
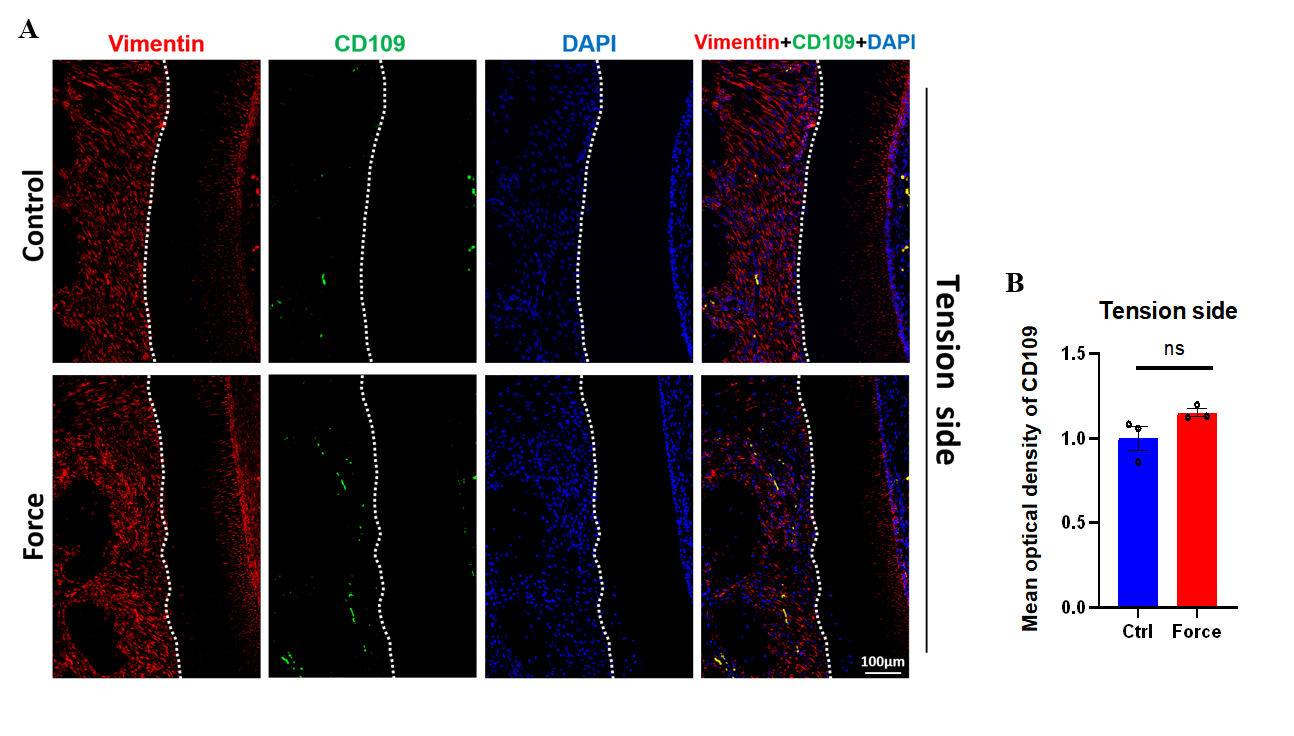
**

**Figure S1** (A-B) Representative immunofluorescence staining and semi-quantifications of CD109 and Vimentin in the tension side of roots. The number of CD109^+^ (green) and Vimentin^+^(red) cells in the Force group showed no significant difference compared with the Control group. N ≥ 3, * P < 0.05; ** P < 0.01; *** P < 0.001. Values are means ± SD.


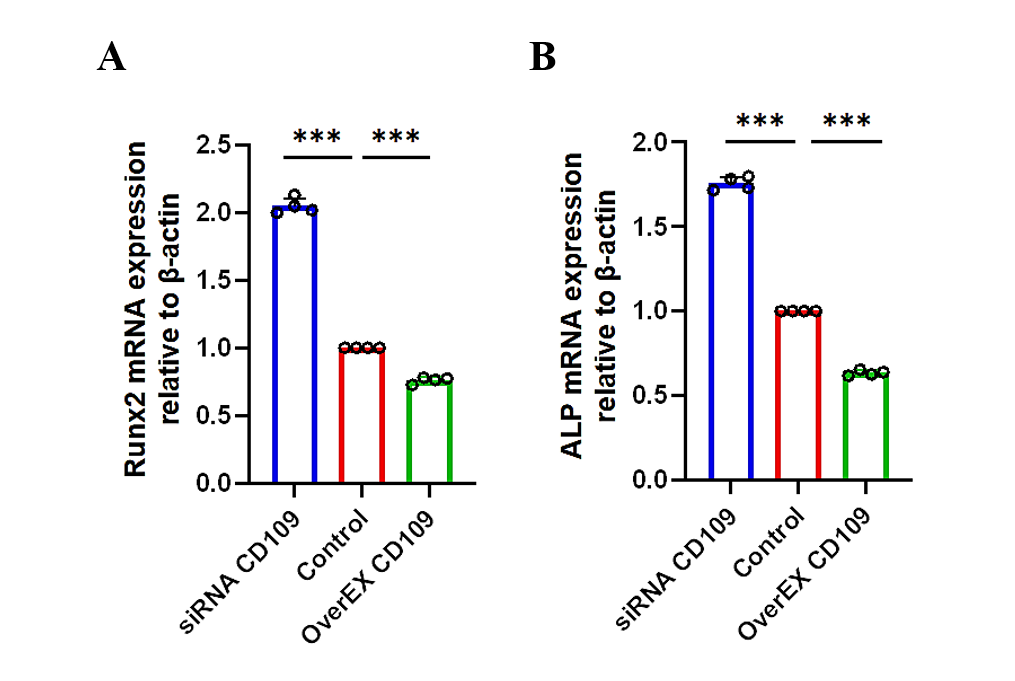


**Figure S2** (A-B) The expression of Runx2, ALP in hPDLSCs after transfections and osteogenic induction for 7 days relative to β-actin by qRT-PCR. The expression of Runx2, ALP mRNA were increased after CD109 being inhibited on hPDLSCs, while decreased after CD109 being overexpressed on hPDLSCs. N ≥  3, * P < 0.05; ** P < 0.01; *** P < 0.001. Values are means ± SD


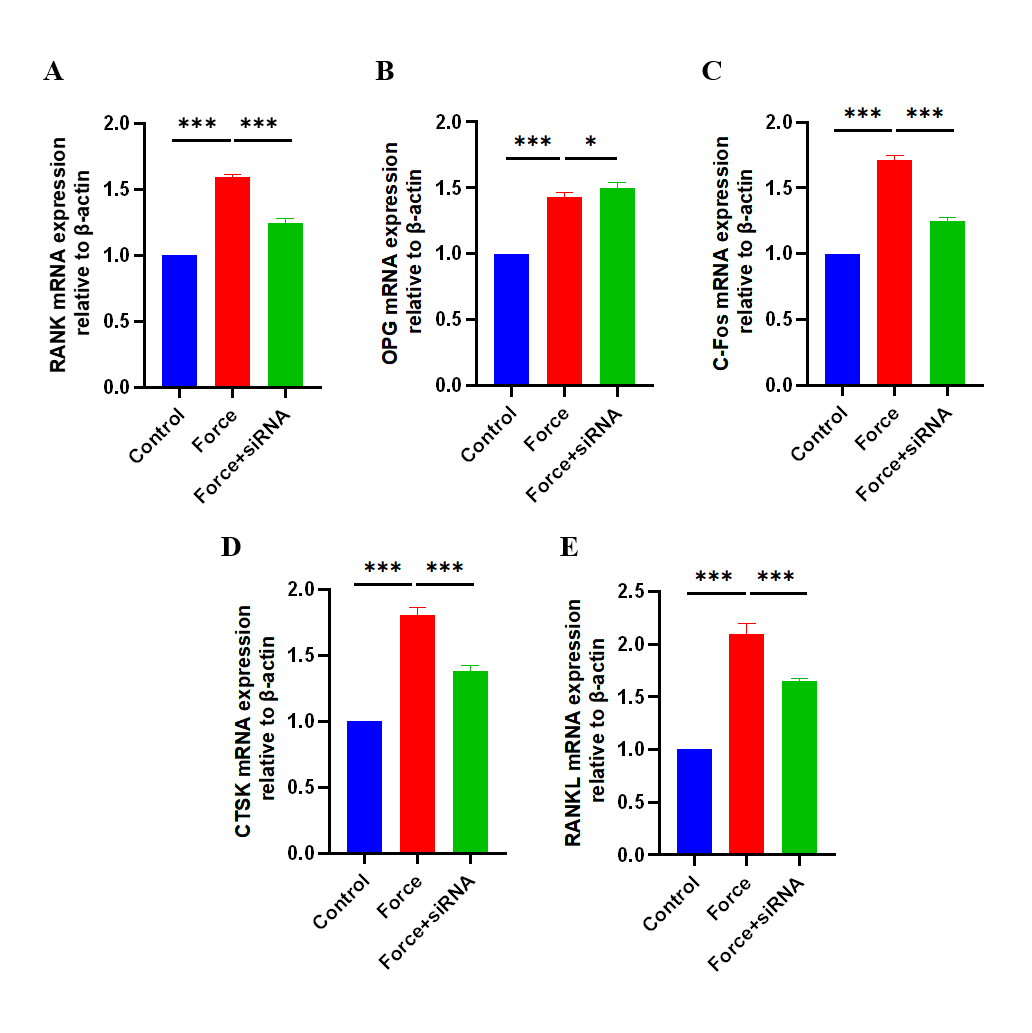


**Figure S3** (A-E) The expression of RANK, CTSK, C-Fos, RANKL and OPG in rBMDMs relative to β-actin on day 7 by qRT-PCR. Conditional medium from Force-PDLSCs enhanced the mRNA expressions of RANK, CTSK, C-Fos, RANKL and OPG, while these effects were partially reversed after transfecting PDLSCs with CD109 siRNA compared with the control. β-actin served as the internal control for equal loading. N ≥ 3, * P < 0.05; ** P < 0.01; *** P < 0.001. Values are means ± SD.


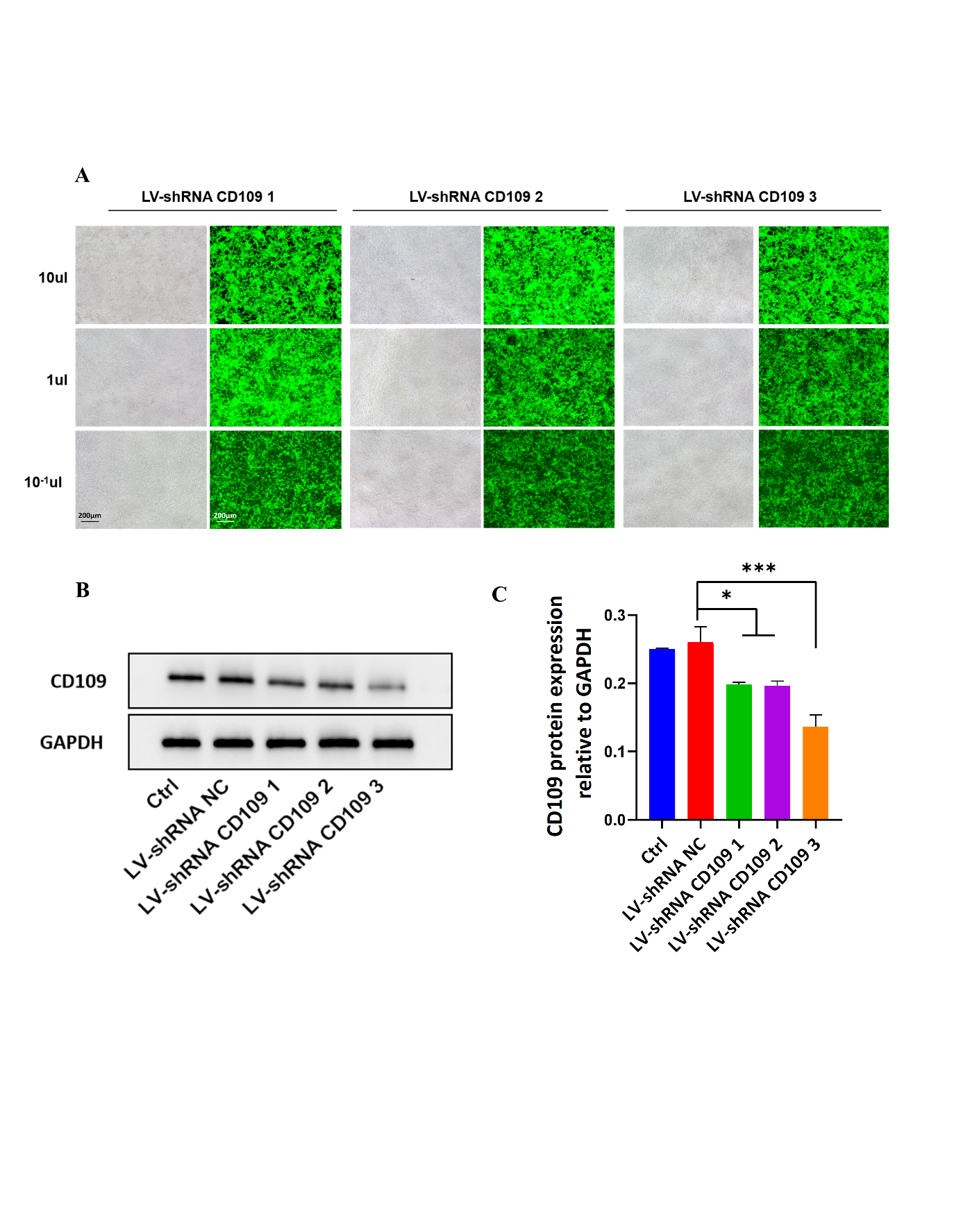


**Figure S4** (A) Representative confocal immunofluorescence images showing LV-shRNA CD109 infected 293T cell to detect the virus titer. (B-C) Western blot results and semi-quantifications of CD109 protein levels in LV-shRNA CD109 3-infect-rat-PDLs were detected by Western blot. LV-shRNA-CD109-3 exhibited the highest silencing efficiency. N ≥ 3, * P < 0.05; ** P < 0.01; *** P < 0.001. Values are means ± SD.


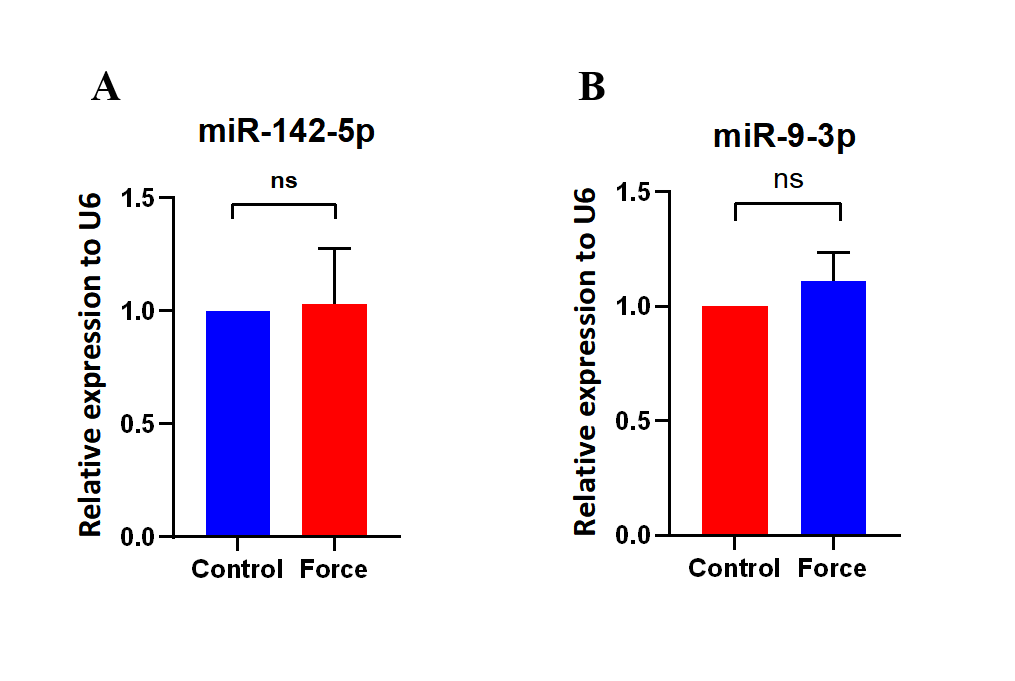


**Figure S5** (A-B) Representative miR-142-5p and miR-9-3p on hPDLSCs relative to U6 by qRT-PCR. Expression of miR-142-5p and miR-9-3p in Force group showed no significant difference compared with the Control group. N ≥ 3, * P < 0.05; ** P < 0.01; *** P < 0.001. Values are means ± SD.
